# Remedies for Sleeplessness

**Published:** 1882-08-20

**Authors:** W. E. Green

**Affiliations:** M. R. C. S., Eng.


### REMEDIES FOR SLEEPLESSNESS.

BY W. E. GREEN, M. R. C. S., ENG.

Alcohol is of great value in producing sleep. Its first effect is to relieve the mind of sad and gloomy thoughts; its second is that of quickening the action of the heart, which it does by producing vasomotor paralysis. This is a most valuable remedy for the sleeplessness of old people, and of those who suffer from cold extremities after going to bed. Brom pot. Its special advantage lies in its utility where cerebral activity is kept up by far away peripheral irritation, especially where this is connected with the pelvic organs. It may either be given alone, with opium, or choral. It increases the effect of balladonna, hyoscyamus, Indian hemp, ether and chloroform. Its constant use leads to diminished brain activity, and to intellectual lethargy. Cannibis indica produces a pleasing and refreshing sleep, but is so uncertain in its action that it is not much used, and moreover, often requires to be given in very large doses; eight grains being sometimes necessary. Chloral is useful in conditions of vascular excitement, either alone or combined with opium; in all cases of sustained high blood pressure, or where there is distinct pyrexia, it is the most useful remedy. It should not be given in cases where the sleeplessness is caused by worry or brain exhaustion. Where sleeplessness is owing to pain it is inferior to most hypnotics. Croton chloral is more serviceable in cases when pain is combined with the insomnia, but it is necessary to give it in large doses (ʒ i.) Hyoscyamus and lupulin take rank with opium, and are serviceable where this drug or its alkaloids disagree. They are both most useful in cases of albuminuria when chloral cannot be taken. Ether and chloroform in full doses are sometimes of use, as is also a solution of nitro-glycerine, which may be given as a substitute for alcohol. Opium, with its alkaloids, is one of the most useful hypnotics, and especially in those conditions which are associated with pain. When vascular excitement exists, it is better combined with tartar emetic, aconite, or other remedies which depress the circulation. The time for giving it should be carefully chosen, the best being that at which the patient is naturally inclined for repose. It should never be given in cases of chronic insomnia, unassociated with other notable disease.—*Birmingham Med. Rev.*—*N. Y. Med. Abs.*

